# Growth Rate of *Usnea aurantiacoatra* (Jacq.) Bory on Fildes Peninsula, Antarctica and Its Climatic Background

**DOI:** 10.1371/journal.pone.0100735

**Published:** 2014-06-26

**Authors:** Ying Li, Bernd Kromer, Gerd Schukraft, Olaf Bubenzer, Man-Rong Huang, Ze-Min Wang, Lin-Gen Bian, Cheng-Sen Li

**Affiliations:** 1 Institute of Botany, Beijing, China; 2 Heidelberg University, Heidelberg, Germany; 3 Klaus-Tschira-Labor (Curt-Engelhorn-Zentrum Archäometrie gGmbH), Mannheim, Germany; 4 Beijing Museum of Natural History, Beijing, China; 5 Wuhan University, Hubei, China; 6 Chinese Academy of Meteorological Sciences, Beijing, China; Agharkar Research Institute, India

## Abstract

The ages of a fruticose lichen of *Usnea aurantiacoatra* (Jacq.) Bory, from Fildes Peninsula, King George Island, Southwest Antarctic, were determined by radiocarbon (^14^C), and it is 1993–1996 at bottom and 2006–2007 at top of the lichen branch. The growth rates of *U. aurantiacoatra* calculated are 4.3 to 5.5 mm year^−1^ based on its length and ages. The comparisons show that the growth rates of *U. aurantiacoatra* are higher than those of *U. antarctica* (0.4 to 1.1 mm year^−1^). The growth rates of fruticose lichens are always higher, usually >2 mm year^−1^, than those of crustose ones, usually <1 mm year^−1^, in polar areas. A warming trend on Fildes Peninsula is recorded in the period from 1969 to 2010 obviously: the mean annual temperature rose from −2.75 to −1.9°C and the average temperature of summer months from 0.95 to 1.4°C, as well as the average temperature of winter months from −6.75 to −5.5°C. The alteration of lichen growth rates in polar areas may respond to the climatic and environmental changes, and the lichens may act as bio-monitor of natural condition.

## Introduction

Lichens, as a unique organism with dual nature, are composed by fungi (mycobionts) and algae or cyanobacteria (photobionts) in a symbiotic relationship and became the most prominent component in vegetation [1]–[3]. Lichens adapt to the extremely adverse ecological conditions from the warm and wet areas along Equator to the cold land in polar region, occupying the natural substrates of rock, soil, trees, etc. in the different habitats of plains, mountains and deserts. Because of their tenacious vitality, lichens, as the primary bio-pioneer, colonize the de-glaciated landscapes in alpine and polar areas [4]. The growth rate of lichen depends on habitat structure, climatic conditions, nutritional uptake and their metabolism. The most lichen species under enriched environmental conditions shows poor growth [5]–[8], Lichens grow extremely slowly with their relatively longevity, and are useful for dating in investigating landscapes, earthquakes, glaciers and archeological remains [4], [9]. Lichens are also sensitive to air quality, especially to pollutions, and thus become a congenital bio-monitor for environmental changes [10].

The Antarctic is defined geographically as the lands and adjoining ice shelves in the south of latitude 60°S. King George Island (61°50′–62°15′ S, 57°30′–59°00′ W), as the largest island of South Shetland Islands in Antarctic, locates about 120 km from the Antarctic continent and about 1100 km from the southern peak of South American continent [11], [12]. South Shetland Islands, including King George Island, belong to the cold-Antarctic zone [1]. Fildes Peninsula (61°51′–62°15′ S, 57°30′–59°00′ W) locates at the southwestern part of King George Island, with its widths varying from 2 to 5 km and its length of approximately 10 km. The substance of Fildes Peninsula composed mainly of basalt, basaltic andesite, volcaniclastic sedimentary rocks and pyroclastic rocks [13]. It is a cold moist maritime climate in Fildes Peninsula and characterized by mean annual air temperatures of −2.1°C [13] and mean air temperatures of above 0°C for up to four months in summer. Precipitation there ranges between 350 and 500 mm per year, with rainfall occurring mainly in summer [14].

Two species of the fruticose lichen *Usnea*, *U. aurantiacoatra* (Jacq.) Bory and *U. antarctica* Du Rietz, grow on Fildes Peninsula, as the most dominant members of lichens there [15]. The growth rates of fruticose lichens are not easy to be measured in field, in comparison with the crustose and foliose ones [4], [16]. In this work, the basal and terminal parts of a branch of *U. aurantiacoatra* collected from Fildes Peninsula were dated by ^14^C and hence the growth rates of this lichen are estimated to be 4.3 to 5.5 mm year^−1^ and are compared with those of other fruticose lichens, including *U. antarctica*, and also of crustose lichens living mainly in polar areas. The climatic conditions for the growths of *U. aurantiacoatra* and *U. antarctica* are considered in this work.

## Materials and Methods

### Collecting site of *Usnea aurantiacoatra* (Jacq.) Bory

**Figure 1:**
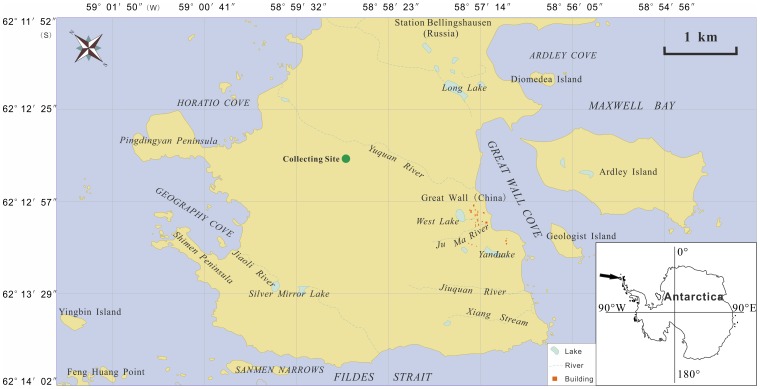
Collecting site of lichen samples in Fildes Peninsula.

The specimen of *U. aurantiacoatra* studied here were collected from Fildes Peninsula, King George Island, Shetland, the southwest Antarctic by the last author C. S. Li during the 23^rd^ Chinese National Antarctica Research Expedition in January 2007 and the specimen collection was permitted by the Chinese Arctic and Antarctic Administration, the State Oceanic Administration, People’s Republic of China. The coordinate of the collecting site is 62°12′42″ S and 58°58′55″ W, with its altitude of 43 m (Fig. 1). The basic substance of the site consists of basalt, and more than 40% land surface of Fildes Peninsula is covered by lichens and mosses.

The collected specimen of *U. aurantiacoatra* was sealed in an airproof plastic bag and put in a box in dark for 3 years until the end of 2009, when it was transported to the laboratory at ETH Zurich in Swiss for dating.

### Description of *Usnea aurantiacoatra* (Jacq.) Bory

Thallus fruticose, 30 to 60 mm high, caespitose, more or less dorsiventral, irregularly dichotomous, richly branched above with abundant attenuate branchlets, main branches obvious. Branches terete, rust red to black at base, yellow-green at other parts, variegate above with wide bands of black pigment, continuously pigmented towards the apices. Surface matt with verrucae, without soredia and isidia. Apothecia abundant, terminal on branches, cupular to bowl-shaped, up to 8 mm in diameters, black, excipulum with numerous verrucae. Pycnidia not seen.

The lichens of *U. aurantiacoatra* are generally upright in growth and widespread on Fildes Peninsula. They are very abundant on the surface of acid and exposed rocks, and also grow in the habitat of mosses [15], [17].

### Dating of *Usnea aurantiacoatra* (Jacq.) Bory

**Figure 2:**
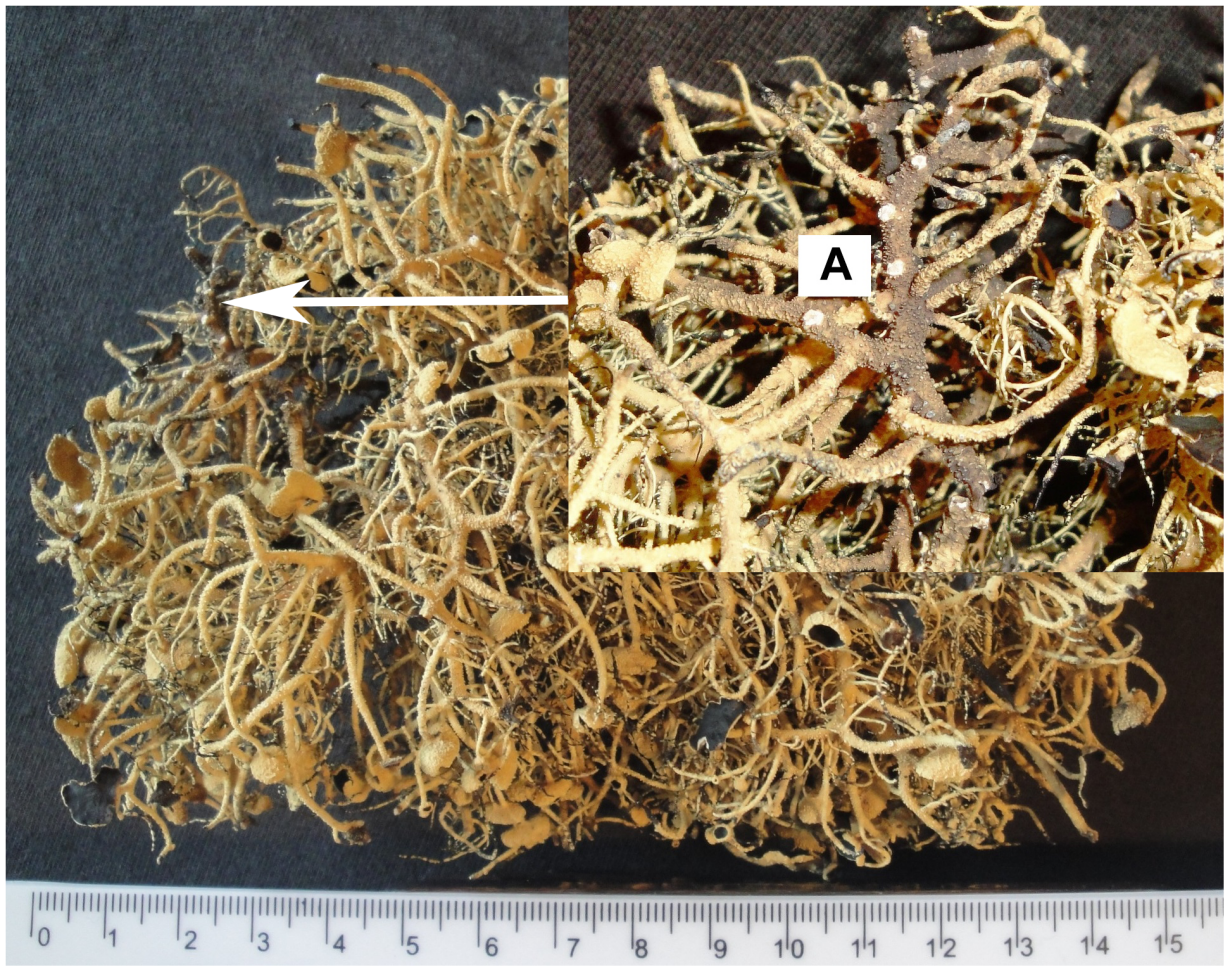
The upper part of *U. aurantiacoatra*, the part which was taken as Sample A.

**Figure 3:**
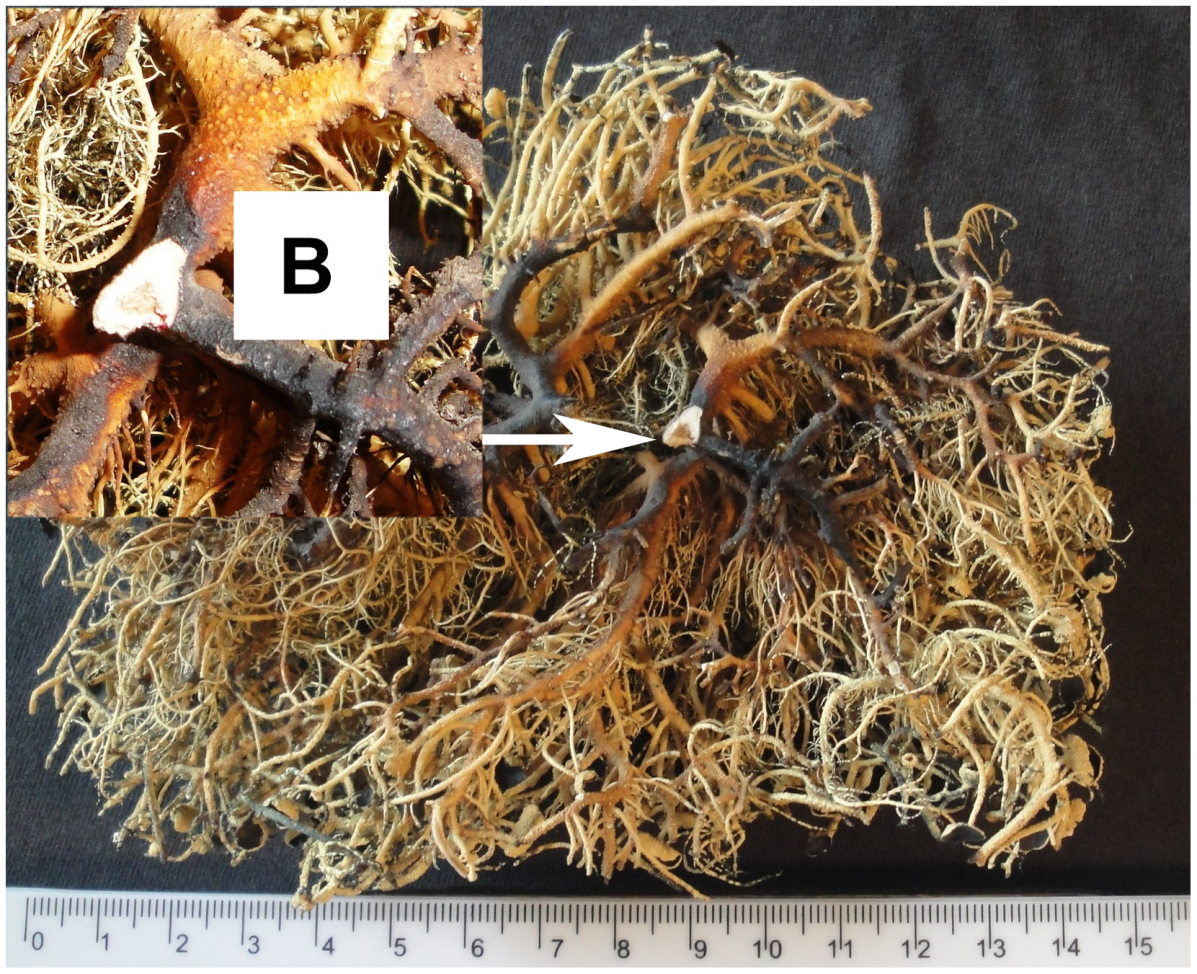
The bottom part of *U. aurantiacoatra*, the part which was taken as Sample B.

Two samples, the top part (named Sample A) and the basal part (Sample B) of a branch of *U. aurantiacoatra*, were taken for dating (Fig. 2 and 3). The top part is an apothecium and the basal is the holdfast attaching to the rock surface.

The sample was dated by AMS-^14^C in the ETH Zurich. The sample was pre-treated by the ABA method (Acid-Base-Acid), i.e. kept in 4% HCl at 60°, 0.4% NaOH at room temperature and 1N HCl at 60° for one hour each step. The insoluble fraction was combusted in an Elemental Analyzer and converted to graphite using Fe as catalyst. ^14^C/^12^C ratios (F^14^C, i.e. the ratio of ^14^C/^12^C in the sample compared to the standard, Reimer at al. 2004) were determined in the MICADAS spectrometer [18].

## Result

### Dating results

#### ^14^C/^12^C ratios

The sample of *U. aurantiacoatra* had F^14^C >1, i.e. it had taken up anthropogenically created ^14^C from the atmospheric bomb testing after 1955, culminating in a doubling of atmospheric ^14^C in 1962/3.

The branch of *U. aurantiacoatra* selected for dating is 60 mm in length between Sample A (Fig. 2) and Sample B (Fig. 3). Sample B was determined to be produced at sometime between 1993 to 1996 after calibration and Sample A was between 2006 to 2007. The estimated age of Sample A (the branch top) coincided with the collection year (2007) of this specimen of *U. aurantiacoatra*, which can be regarded as the time of the stopping growth of the lichen. Therefore, the dating result of *U. aurantiacoatra* is accurate and reliable. Accordingly, the branch ages will fall into the range of 11 (1996 to 2007) to 14 (1993 to 2007) years.

### The growth rates

The ages of this branch are detected to be 11 to 14 years old, and the aged branch is 60 mm in length, the growth rates of the lichen are therefore 4.3 to 5.5 mm year^−1^.

## Discussions

As a pioneer organism, lichens can promote the weathering of rocks and pedogenesis, and create appropriate conditions for the growth of subsequent plants in the harsh environments, like desertification land and polar areas. The weathering action can be a series of physical, chemical or combined processes [19]. Comparing with bryophytes and vascular plants, the growth of lichens is very slow, especially, when they live in the extremely arid and cold habitats, such as Antarctic areas.

### The lichen of *Usnea* on Fildes Peninsula

The vegetation on King George Island is the poorly developed tundra and consists almost exclusively of cryptogams, lichens and mosses, with two spices of flower plants of *Deschampsia antarctica* and *Colobanthus quitensis* [20], [21].

The species of *Neuropogon* group in the genus *Usnea* thrive in Polar Regions and higher altitudes of temperate and tropical regions, and are often the dominant fruticose lichens in such harsh environments [22]–[24]. During the 9^th^ and 11^th^ Chinese National Antarctica Research Expeditions, Chen [15] collected and indentified two species of this group, *U. antarctica* Du Rietz and *U. aurantiacoatra* (Jacq.) Bory, on Fildes Peninsula, and demonstrated that *U. aurantiacoatra* dominates in the local lichen vegetation.

### Comparison of the growth rates of *Usnea aurantiacoatra* (Jacq.) Bory with those of *Usnea antarctica* Du Rietz

The growth rate of *U. antarctica* was estimated based on the investigation of the lichens on Ardley Island close to Fildes Peninsula [15]. The thalli of *U. antarctica* grew on the wood block of tripods there, which was built for mapping by the staff of the station of former Soviet Union in 1970, and measured to be 7 to 20 mm in length in 1993 [15]. In general, the fresh wood block was not suitable for the lichen to grow on it at beginning years, and it is assumed that the lichens colonized the block at least 5 years later. That means the thalli of *U. antarctica* produced their length of 7 to 20 mm there in 18 years (from 1975 to 1993), corresponding to a growth rate of 0.4 to 1.1 mm year^−1^ [15].

Another example of the growth rate of *U. antarctica* was provided on a moraine (62°39′S, 60°23′W, 125 m asl), which is close to the Spanish Antarctic Base Juan Carlos I in South Bay, Livingston Island, South Shetland Islands, and 1 km from the coast. Ten boulders with different sizes were selected in 1991 and were marked in two designed experimental zones. Growing upright on the boulders, the thalli of *U. antarctica* were measured in 1991 and re-measured in January 2002. The lichens grew for 22 mm in length in 11 years (Table 1 in [4]), and their growth rates were calculated to be 2.0 mm year^−1^ [4].

**Table 1:** Growth rates of fruticose lichens (^*1^: [25], ^*2^: [27], ^*3^: [26], ^*4^: [28], ^*5^: [29], ^*6^: [7], [30]–[32], ^*7^: [33], ^*8^: [34] cited in [35], ^*9^: [36] cited in [7], [32]).

Average annual growth rate (mm year^−1^)	
*Cladonia alpestris* ^*1,2^	3.3–5.8
*Cladonia rangiferna* ^*1,2,3,4^	2.7–5.6
*Cladonia sylvatica* ^*1^	5.2–5.5
*Cladonia mitis* ^*3,5^	3.0–5.2
*Cladonia coccifera* ^*6^	1.6–2.0
*Evernia prunastri* ^*7^	2.0
*Bryoria* spp. ^*8^	13.8
*Ramalina reticulata* ( = *R. menziesii*)^ *9^	30.0 (11.0–90.0)

Comparing with the growth rates of *U. antarctica* (0.4 to 1.1 mm, or 2.0 mm year^−1^), the rates of *U. aurantiacoatra* are much higher (4.3 to 5.5 mm year^−1^). The cause of the different rates of these two species of *Usnea* may be intrinsic. In Fildes Peninsula, *U. antarctica* appears at regions adjacent to the coast. When occasionally growing together with *U. aurantiacoatra*, it often occurs at the margin of the population of the latter [15]. Therefore, it seems that *U. antactica* is less competitive than *U. aurantiacoatra*.

### Comparing the growth rates of *U. aurantiacoatra* (Jacq.) Bory with those of other fruticose lichens

The linear growth rates of *Cladonia rangiferina* (L.) Weber ex F.H. Wigg. varied in different places from the North to South Hemispheres: its rates are 5.0 to 5.6 mm year^−1^ in spruce forest on Seward Peninsula, Alaska [25], 3.9 to 4.3 mm year^−1^ at the site from Alakitka situated in Kuusamo, northeastern Finland [26], 2.7 mm year^−1^ in tundra communities on Chukotsk Peninsula, the western Siberia [27], and 4.65 to 5.33 mm year^−1^ on the sub-Antarctic island of South Georgia [28].

The growth rates of *C. alpestris* (L.) Rabenh. were different in two localities: they are 4.3 to 5.8 mm year^−1^ on the Seward Peninsula, Alaska [25] and 3.3 mm year^−1^ on Chukotsk Peninsula, the western Siberia [27]. The rates of *C. sylvatica* living on the Seward Peninsula, Alaska are 5.2 to 5.5 mm year^−1^ [25].

The growth rates of *C. mitis* Sandst. varied from 3.0 to 3.5 mm year^−1^ at Alakitka situated in Kuusamo, northeastern Finland [26] to 5.2 mm year^−1^ at Pine Barrens near Mauston, Juneau County, Wisconsin, USA [29].

Considering the collected data of fruticose lichens (Table 1), the rates of *U. aurantiacoatra*, 4.3 to 5.5 mm year^−1^, are more or less similar to those of *C. alpestris* (3.3–5.8 mm year^−1^), *C. rangiferina* (2.7–5.6 mm year^−1^), *C. sylvatica* (5.2–5.6 mm year^−1^) and *C. mitis* (3.0–5.2 mm year^−1^ ), but higher than those of *C. coccifera* (1.6 to 2.0 mm year^−1^) and *Evernia prunastri* (L.) Ach. (2.0 mm year^−1^). Comparing with *Bryoria* spp. (rate: 13.8 mm year^−1^) and *Ramalina reticulata* (Nohden) Krempelh ( = *R. menziesii* Taylor) (rate: 30 mm year^−1^), *U. aurantiacoatra* grows more slowly.

### Comparing the growth rates of fruticose lichens with those of crustose ones in polar areas

The growths of crustose lichens were investigated mainly in the temperate and Arctic areas for dating attached substrata [37]–[43]. The annual radial growth rates of crustose lichens are usually <0.5 mm year^−1^, for example, the lichen of *Rhizocarpon geographicum* (L.) DC. living in alpine area has a growth rate below 0.5 mm year^−1^ [42]. But some exceptional species, like *Aspicilia alphoplaca* (Wahlenb.) Poelt & Leuckert and *Trapelia coarctata* (Turner ex Sm.) M. Choisy, may expand 1 mm year^−1^ [4], [42].

The rates of *Acarospora macrocyclos* Vain. are 1.0–1.2 mm year^−1^ (>1 mm year^−1^) in Signy and Livingston Islands, Antarctic, and it is the high rate in the crustose (Placodioid) lichens in polar areas [4], [44], [45].

The growth rates of most known crustose lichens in polar areas are usually less than 1 mm year^−1^. For example, the rates of *Rhizocarpon geographicum* are 0.025–0.5 mm year^−1^ in Signy and Livingston Islands, and 0.1–1.4 mm year^−1^ in Antarctic and Arctic [3], [4], [45]–[47]. The rates of *Caloplaca sublobulata* (Nyl.) Zahlbr. are 0.82–0.95 mm year^−1^ in Livingston Island [4], [47]. The rates of *Buellia frigida* Darb. are 0.01–0.07 mm year^−1^ in Dry Valleys, Cape Hallett [3], and those of *Buellia latemarginata* Darb. are 0.5–0.87 mm year^−1^ in Signy and Livingston Island [3], [4]. The rates of *Bellemerea* sp. are 0.75 mm year^−1^ in Livingston Island [4].

The growth rates of fruticose lichens are always higher (usually >2 mm year^−1^; Table 1) than those of crustose ones (usually <1 mm year^−1^; Table 2) in polar areas. Only the rates of *U. antarctica*, with 0.4 to 1.1 mm year^−1^, on Ardley Island are close to those of crustose lichens.

**Table 2:** Growth rates of crustose lichens in polar areas (^*1^: [44], ^*2^: [45], [46], ^*3^: [47], ^*4^: [3], ^*5^: [4]).

Average annual growth rate (mm year^−1^)	
*Acarospora macrocyclos* ^*1,2,5^	1.0–1.2
*Rhizocarpon geographicum* ^*2,3,4,5^	0.025–0.50.1–1.4
*Caloplaca sublobulata* ^*3,5^	0.82–0.95
*Buellia latemarginata* ^*4,5^	0.5–0.87
*Buellia frigid* ^*4^	0.01–0.07
*Bellemerea* sp.^*5^	0.75

The two kinds of lichens, fruticose and crustose ones, possess the different habits and biological features. The former with a holdfast attach to the surface of substance (such as rocks) and grow as upright or pendulous branches in three-dimensions with its increasing linear length, while the latter creep on the surface and expand in two-dimensions with its increasing diameter. Perhaps, the fruticose lichens may obtain more light in 3-D growth forms for their photosynthesis than the crustose lichens in a 2-D pattern.

### The climatic background of growths of *Usnea* on Fildes Peninsula

The lichen growths are influenced by the environmental factors, including light, temperature, precipitation, attached substance, and the interaction with other organisms at immediate vicinity. In this part, we consider the temperature data of Fildes Peninsula in relation to the growths of lichens of *U. aurantiacoatra* and *U. antarctica*.

### Climatic Changes on Fildes Peninsula – Warming Period of 1969–2010

The Little Ice Age is about from 1300 to 1870 [48]. Europe, North America, and Asia faced much colder winter than usual. Mountain glaciers, such as the glaciers in the Alps, Norway, Ireland, and Alaska, were expanded rapidly. During this period, there were three maxima, beginning about 1650, about 1770, and 1850, each separated by slight warming intervals [49]. And in Antarctic, it had also a cooling period in the late 1700s and 1800s and then a warming period over the 19th century. It is in close fit with the Little Ice Age. It records the overall warming at permanently occupied stations on the Antarctic continent (data from 1959 to 1996) and Southern Ocean Island stations (data from 1949 to 1996). The 16 Antarctic Stations have recorded a warming trend with a mean rate of 0.9–1.2°C per century (0.009–0.012°C year^−1^), and the 22 Southern Ocean Stations have recorded a mean rate of 0.7–1.0°C per century (0.007–0.010°C year^−1^). Antarctic Peninsula stations show a consistent regional rate of warming that is more than twice than the average of other Antarctic Stations [50].

We collected meteorological data available from Station Bellingshausen, Russia (SBR, 62°11′47′′S, 58°57′39′′W, 15.4 m asl, No. 89050) [51], [52] and from Great Wall Station, P. R. China (GWSC, 62°12′59′′S, 58°57′52′′W, 10 m asl, No. 89058) [53], [54] on Fildes Peninsula. The two stations are separated by a distance of 2.1 km on the same peninsula.

**Figure 4:**
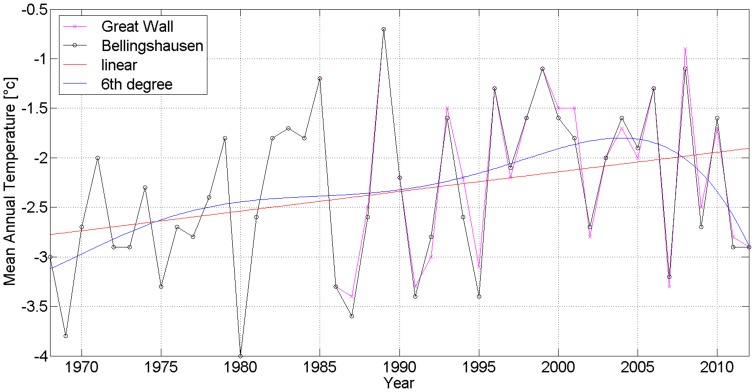
The mean annual temperature at Station Bellingshausen and Great Wall Station on Fildes Peninsula, Antarctica, by using MATLAB and calculating with linear and 6^th^ degree [55], [56].

**Figure 5:**
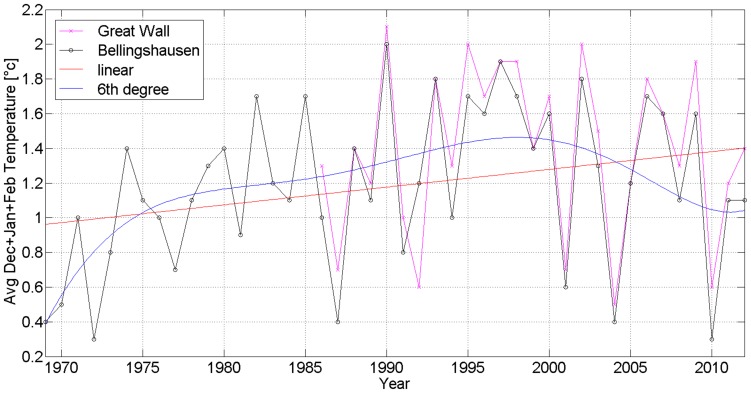
The average summer monthly temperature (December, January and February) at Station Bellingshausen and Great Wall Station on Fildes Peninsula, Antarctica, by using MATLAB and calculating with linear and 6^th^ degree [55], [56].

**Figure 6:**
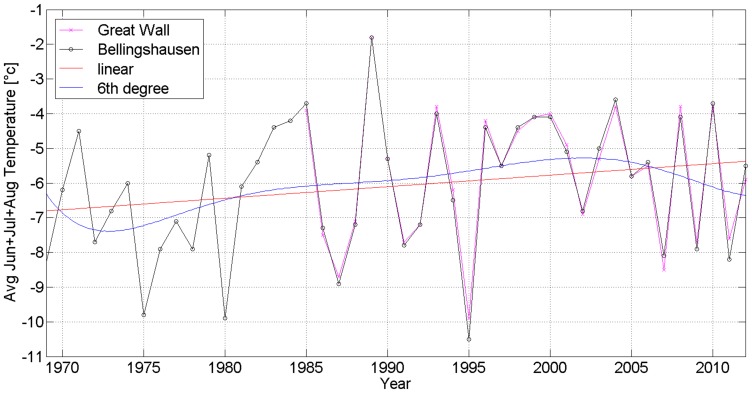
The average winter monthly temperature (June, July and August) at Station Bellingshausen and Great Wall Station on Fildes Peninsula, Antarctica, by using MATLAB and calculating with linear and 6^th^ degree [55], [56].

The mean annual temperature (MAT) is −2.3°C at SBR from 1969 to 2010 and −2.1°C at GWSC from 1985 to 2012. The average summer monthly temperature (ASMT in January and February) is 1.6°C at SBR from 1969 to 2010 and 1.8°C at GWSC from 1985 to 2010. The average winter monthly temperature (AWMT in July and August) is −6.4°C at SBR from 1969 to 2010 and −5.9°C at GWSC from 1985 to 2010 (Fig. 4, 5 and 6) [55], [56]. So the difference of MATs and ASMTs of two stations is about 0.2°C, and the difference of AWMTs of two stations is about 0.5°C.

The MATs of two stations have been rising from −2.75 to −1.9°C in the period from 1969 to 2010 (Fig. 4, red line) and the ASMTs (December, January and February) rising from 0.95 to 1.4°C (Fig. 5, red line), as well as the AWMTs (June, July and August) rising from −6.75 to −5.5°C (Fig. 6, red line). Therefore, an obvious warming trend in Fildes Peninsula was recorded in these two stations, with the temperatures rising higher in winter than in summer (0.030 vs 0.011°C year^−1^) in 41 years (Table 3).

**Table 3:** The temperature changes on Fildes Peninsula in 41 years.

Temperature (°C) changes in periods of 41 years (1969–2010)			
Mean Annual Temperature	−2.75 to −1.9	0.85↑	0.021↑year^−1^
Average Summer Monthly Temperature	0.95 to1.4	0.45↑	0.011↑year^−1^
Average Winter Monthly Temperature	−6.75 to −5.5	1.25↑	0.030↑year^−1^

An increase of the mean surface temperature of 1–5°C is a key prediction of climate change in the Antarctic, but at a lower rate than in the Arctic, and precipitation is expected to increase by up to 30% across the continental Antarctica [57]. The global warming resulted in a global increase of temperature of 0.03°C year^−1^ (0.02–0.05°C year^−1^ range) is predicted to be 0.5–0.7 times larger in maritime Antarctica [3]. The mean annual temperature rose 0.021°C year^−1^ on Fildes Peninsula (62°S) in 41 years (in this work), less than 0.056°C year^−1^ at Faraday Station (65°S) in 45 years [3], both in Antarctic Peninsula. As predicted by global change models, the rise has been particularly large in winter temperatures, with the large interannual variability [58]. In our work, it proves that the temperature rise was **higher in winter than in summer** (0.030 vs 0.011year^−1^) on Fildes Peninsula in the last 41 years.

The lichens grow in Antarctic mainly controlled by two factors, temperature and precipitation. When the two factors changed, the lichen growth would be influenced significantly. For example, the radial growth of *Buellia frigida* became fast with the increase of growth rate from 0.01 to 0.07 mm year^−1^, when the mean temperatures rose from −4.8 to −1.4°C in summer and −30.5 to −26.4°C in winter, with the precipitation increase from 50 to 225 mm in rainfall equivalent. The same things happened with *Buellia latemarginata*, whose growth rates increased from 0.5 to 0.87 mm year^−1^ responding to the precipitation increases from 400 to 800 mm, with the stable summer temperature (1.3 to 1.3°C) and the rising winter temperature from −9.0 to −7.0°C [3].

The different growth rates of *U. aurantiacoatra* and *U. antarctica* (4.3 to 5.5 vs. 0.4 to 1.1 mm year^−1^) on Fildes Peninsula may mainly attribute to their different biological features, as mentioned before. The different attached substances of these lichens, rock vs. the wood block, may also played an important role in their growth rate. The temperature changes during the years may also influence the growth of the two species.

### Climatic Change on Antarctic – Short Cooling Period of 1979–1998

When we consider the warming period over the 19th century in Antarctic, on another hand, it has been slightly cooled from 1979 to 1998 [59]. Doran [58] also pointed out that a seasonally averaged surface air temperature in the Dry Valleys of continental Antarctica was decreased by 0.7°C per decade (0.07°C year^−1^) from 1986 to 1999, especially cooling of 1.2°C per decade (0.12°C year^−1^) in summer.

The growth rates of *U. antarctica* (from 0.4 to 1.1 mm year^−1^) were obtained according to the assumed growth period between 1975 and 1993 [15], with a 14-year duration falling into this cooling interval (1979 to 1993). By comparison, the growth of *U. aurantiacoatra* analyzed here with its rates of 4.3 to 5.5 mm year^−1^ was in the period between 1993 and January 2007, with a 5-year (1993 to 1998) duration falling into the cooling interval, shorter than that of *U. antarctica*. This fact may also influence the different growth rates of these two lichens in some extents.

## Conclusion

1. By ^14^C dating, a branch of *Usnea aurantiacoatra* (Jacq.) Bory was detected to be 1993–1996 at basal part and 2006–2007 at tip in ages. The latter estimated age of 2007 coincides with the collection year (2007) of this specimen in January of 2007, when the lichen stopped its growth. So, the dating result is accurate and reliable.
2. Based on the length (60 mm) and ages (11 to 14 years) of this branch, the growth rates of *U. aurantiacoatra*, found attached to the rocks in situ on Fildes Peninsula, were calculated to be 4.3 to 5.5 mm year^−1^. The rates of *U. antarctica* Du Rietz, grew on introduced substrata (wood block) on Ardley Island, were estimated to be 0.4 to 1.1 mm year^−1^ previously.
3. The growth rates of fruticose lichens are always higher, usually >2 mm year^−1^, than those of crustose lichens, usually <1 mm year^−1^, in polar areas.
4. The mean annual temperature have been rising from −2.75 to −1.9°C in the period on Fildes Peninsula from 1969 to 2010, and the average summer monthly temperature (December–February) rising from 0.95 to 1.4°C, as well as the average winter monthly temperature (June–August) rising from −6.75 to −5.5°C. A warming trend on Fildes Peninsula is obvious, especially with the temperatures rising higher in winter than in summer (0.030 vs 0.011°C year^−1^).
5. The climate and environment changes influence the lichen growths in polar areas significantly. When the ages of lichens are detected exactly, the changes of lichen growth in these ages may respond to the climate and environment changes, and the lichens may play a bio-monitor of natural condition therefore.
